# Curcumin attenuates angiotensin II‐induced podocyte injury and apoptosis by inhibiting endoplasmic reticulum stress

**DOI:** 10.1002/2211-5463.12946

**Published:** 2020-08-28

**Authors:** Nan Yu, Lin Yang, Lilu Ling, Yuan Liu, Ying Yu, Qing Wu, Yong Gu, Jianying Niu

**Affiliations:** ^1^ Department of Nephrology Shanghai Fifth People's Hospital Fudan University Shanghai China; ^2^ Department of Nephrology Huashan Hospital Fudan University Shanghai China

**Keywords:** angiotensin II, CKD, curcumin, ER stress, podocyte

## Abstract

Podocytes are an important component of the glomerular filtration barrier in the kidneys. The dysfunction and apoptosis of podocytes are important factors that can lead to the progression of chronic kidney disease (CKD). In CKD, angiotensin II is continuously elevated in circulation and is considered to have key roles in inducing podocyte injury and apoptosis. Curcumin is a hydrophobic polyphenolic compound extracted from turmeric. Increasing evidence demonstrates that curcumin has a protective effect on the kidneys in CKD. However, the mechanisms mediating this protective effect remain unclear. The aim of this study was to explore whether curcumin could protect against angiotensin II‐induced injury and apoptosis of podocytes. We performed western blotting, immunofluorescence, phalloidin staining, and terminal deoxynucleotidyl transferase nick‐end labeling staining to observe the expression level of podocyte‐specific proteins, apoptosis‐related proteins, and the arrangement of F‐actin. We found that curcumin could reverse angiotensin II‐induced podocyte injury and apoptosis in a dose‐dependent manner. In addition, curcumin dose‐dependently attenuated a pro‐apoptotic pathway, activated by angiotensin II‐induced endoplasmic reticulum stress. Conversely, the protective effects of curcumin were impaired upon addition of tunicamycin, an activator of endoplasmic reticulum stress. Thus, we speculate that curcumin protects against angiotensin II‐induced podocyte injury and apoptosis, at least partly by inhibiting endoplasmic reticulum stress.

## Graphical abstract

Here, we show that curcumin, an active hydrophobic polyphenolic compound extracted from turmeric, could reverse angiotensin II‐induced podocyte injury and apoptosis in a dose‐dependent manner. We found this protective effect of curcumin to be at least partly achieved by inhibiting endoplasmic reticulum stress. Therefore, curcumin may have potential for future development into a possible treatment for chronic kidney disease.

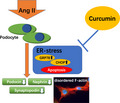

**Abbreviations**
Ang II: angiotensin II
AT1R: angiotensin II receptor
ATF4: activating transcription factor 4
CHOP: C/EBP homologous protein
CKD: chronic kidney disease
eIF2α: eukaryotic initiation factor
ER stress: endoplasmic reticulum stress
GRP78: glucose‐regulated protein, 78kDa
RAS: renin–angiotensin system
TRITC: tetramethylrhodamine
TUNEL: terminal deoxynucleotidyl transferase nick‐end labeling
WT1: Wilms' tumor 1

Podocytes, one of the components that constitute the glomerular filtration barrier, are terminally differentiated and highly branched glomerular visceral epithelial cells [1]. Podocyte dysfunction leads to proteinuria and plays a role in the initiation of glomerular diseases and the development of renal failure [2, 3]. Angiotensin II (Ang II), a key molecule of the renin–angiotensin system (RAS), is a significant risk factor for the initiation and progression of chronic kidney disease (CKD). Increasing evidence shows that Ang II might induce podocyte foot process fusion and apoptosis, and accelerates the progression to end‐stage renal disease [4]. However, the mechanism of Ang II‐induced podocyte apoptosis has not been clarified.

Curcumin is an active hydrophobic polyphenolic compound extracted from turmeric [5] that is frequently used in Asia as a fragrance, coloring, and additive. Curcumin is considered a therapeutic agent in traditional medicine in India and China. Recently, curcumin has been shown to have antitumor [6], anti‐inflammatory, antioxidation, hypoglycemic, lipid‐lowering, and analgesic effects [7, 8, 9], providing potential therapeutic value in numerous diseases [9]. Earlier studies determined that curcumin could improve renal function and renal tissue fibrosis in CKD animal models [8, 10]. However, the mechanisms underlying the protective effects of curcumin on the kidney in CKD remain unclear.

Ang II can induce endoplasmic reticulum stress (ER stress) and apoptosis via the angiotensin II receptor (AT1R) [11]. Elevated levels of Ang II in the kidney could induce ER stress, which activates unfolded protein responses and apoptosis [12]. A certain degree of ER stress can be restored by self‐regulation. Prolonged ER stress causes activation of protein kinase‐like ER kinase and phosphorylation of eukaryotic initiation factor (eIF2α), leading to activation of transcription factor 4 (ATF4) and the transcription factor C/EBP homologous protein (CHOP). CHOP can upregulate apoptotic Bax (Bcl‐2‐associated X, apoptosis regulator) and downregulate anti‐apoptotic Bcl‐2 (Bcl‐2 apoptosis regulator), thus initiating cell apoptosis [13, 14]. Previous evidence showed that activated ER stress in the kidney leads to podocyte apoptosis and renal injury [15, 16, 17, 18]. Thus, the present study aimed to investigate whether curcumin could protect against podocyte injury and apoptosis by inhibiting Ang II‐activated ER stress.

## Materials and methods

### Reagents and antibodies

Advanced Roswell Park Memorial Institute (RPMI) 1640 medium and fetal bovine serum were provided by Gibco (Gaithersburg, MD, USA). Curcumin was purchased from Fluka (Mexico City, Mexico), Ang II was purchased from Sigma‐Aldrich (St. Louis, MO, USA), Tetramethylrhodamine (TRITC)–phalloidin was purchased from YEASEN (Shanghai, China). Cell Counting Kit‐8 (CCK‐8) was purchased from Dojindo (Kumamoto, Japan). Antibodies against β‐actin, glucose‐regulated protein, 78kDa (GRP78), CHOP, caspase‐3, ATF4, eIF2a, and phosphorylated (p)‐eIF2a were purchased from Cell Signaling Technology, Inc (CST, Danvers, MA, USA). Antibodies against nephrin, podocin, and synaptopodin were from Abcam (Cambridge, UK). Antibodies against WT1, Bax, and Bcl‐2 were obtained from Santa Cruz, Inc (Santa Cruz, CA, USA). Fluorescein (FITC)‐AffiniPure goat anti‐mouse and goat anti‐rabbit secondary antibodies were purchased from the Jackson Laboratory (Bar Harbor, ME, USA). 2‐(4‐Amidinophenyl)‐1H‐indole‐6‐carboxamidine (DAPI) was purchased from Beyotime (Shanghai, China). A terminal deoxynucleotidyl transferase nick‐end labeling (TUNEL) assay kit was purchased from Roche Diagnostics (Mannheim, Germany).

### Cell culture and treatment

Conditionally immortalized mouse podocytes (MPC5) were kindly donated by C. Hao (Huashan Hospital affiliated to Fudan University, Shanghai, China). Podocytes were cultured in RPMI‐1640 medium supplemented with 10% FBS, penicillin (100 U·mL^−1^), and streptomycin (100 mg·mL^−1^) with 5% CO_2_ at 33 °C for proliferation and then cultured at 37 °C for 10–14 days without interferon‐γ for differentiation. The medium was exchanged as appropriate. Cells at 80% confluence were growth‐arrested using serum starvation overnight. Differentiated podocytes were treated with or without Ang II at a final concentration of 1 × 10^−6^ mol·L^−1^ for 48 h. As indicated, the cells were pretreated with curcumin (1, 5, and 10 μm) for 1 h. The concentrations of Ang II and tunicamycin were selected based on our previous studies [12].

### CCK‐8 assay

The CCK‐8 Kit was applied to evaluate viability of the podocytes. Cells were resuspended and seeded into 96‐well plates at a density of 5 × 10^3^ cells/well with three replicates. After 12 h, the cells were treated with curcumin in different concentrations (1–20 μm) for 48 h. Subsequently, CCK‐8 was added to each well and further incubated at 37 °C for 1 h. The absorbance of CCK‐8 was detected at 450 nm using a Tecan Infinite 200 Pro multiwell plate reader (Männedorf, Switzerland). The experiment was performed in triplicate.

### Immunofluorescence

Immunofluorescence staining was performed on cells using a standard protocol. Briefly, after three washes with ice‐cold PBS, the cells were fixed with 4% paraformaldehyde for 30 min at room temperature and permeabilized using 0.1% Triton X‐100 for 15 min. After blocking with 5% bovine serum albumin in PBS for 60 min at 37 °C, the cells were stained with the appropriate primary antibody dilutions [anti‐podocin (1 : 200)] at 4 °C overnight. After washing three times, the cells were incubated with Fluorescein (FITC)‐AffiniPure goat anti‐rabbit secondary antibodies for 60 min at room temperature. Cell nuclei were stained with DAPI for 5 min at room temperature. Fluorescence images were immediately captured under fluorescence microscopy (Olympus, Tokyo, Japan).

### Western blotting analysis

The treated cells were washed thrice with ice‐cold PBS before being lysed with radioimmunoprecipitation assay buffer containing a protease and phosphorylase inhibitor on ice for 30 min. Total proteins were obtained by centrifugation for 15 min (13 523 ***g***, 4 °C). The protein concentration was quantified using a Bicinchoninic Acid Protein Assay kit according to the manufacturer's instructions (Thermo Fisher Scientific, Waltham, MA, USA). Then, 20 μg protein samples were subjected to SDS/PAGE and then transferred onto polyvinylidene fluoride membranes (Millipore, Burlington, MA, USA). After blocking with 5% nonfat dried milk for 2 h at room temperature, the membranes were incubated with primary antibodies against β‐actin (1 : 1000), GRP78 (1 : 1000), CHOP (1 : 1000), caspase‐3 (1 : 1000), LC3 (1 : 500), P62 (1 : 1000), ATF4 (1 : 500), eIF2a (1 : 1000), p‐eIF2a (1 : 500), CHOP (1 : 1000), nephrin (1 : 500), podocin (1 : 1000), Bax (1 : 500), and Bcl‐2 (1 : 1000) at 4 °C overnight. The membranes were washed three times in Tris‐buffered saline/Tween‐20 and then incubated with horseradish peroxidase‐labeled secondary antibodies (CST) for 1.5 h at room temperature. Protein bands were visualized using enhanced chemiluminescence (Share‐bio, Qianjiang, China). Each experiment was conducted in triplicate. The signals were analyzed using the imagej software (NIH, Bethesda, MD, USA).

### Phalloidin staining

Phalloidin staining was performed using a standard protocol. Briefly, after washing three times with ice‐cold PBS, the cells were fixed with 4% paraformaldehyde for 30 min at room temperature and permeabilized using 0.1% Triton X‐100 for 15 min. The sample was washed three times with PBS before incubation with primary antibody dilutions [anti‐WT1 (1 : 100)] and appropriate secondary antibodies. Then, the cells were incubated with 200 μL 1% BSA TRITC–phalloidin working fluid in the dark for 30 min at room temperature. After washing three times, cell nuclei were stained with DAPI for 5 min at room temperature. Images were immediately collected under a fluorescence microscope (Olympus).

### TUNEL staining

An *In Situ* Cell Death Detection Kit was used to detect cell apoptosis according to the manufacturer's instructions. MPC5 cells were seeded in 24‐well plates and then cultured with Ang II and curcumin. 48 h later, the cells were fixed with 4% paraformaldehyde for 30 min at room temperature and permeabilized using 0.1% Triton X‐100 for 2 min on ice. The cells were washed three times with PBS and incubated with the TUNEL reaction mixture at 37 °C for 1 h in the dark. After counterstaining the nuclei with DAPI, the samples were examined using fluorescence microscopy (Olympus).

### Statistical analyses

Quantitative data are reported as the mean ± standard deviation (SD). One‐way analysis of variance (ANOVA) was performed to compare data from more than two groups, while Tukey's test was used to analyze significant differences between two groups. Statistical analyses were performed using graphpad prism for Mac (version 7; GraphPad Inc., La Jolla, CA, USA). *P*‐values less than 0.05 were considered statistically significant in all tests.

## Results

### Effect of curcumin treatment on podocyte viability

**Fig. 1:**
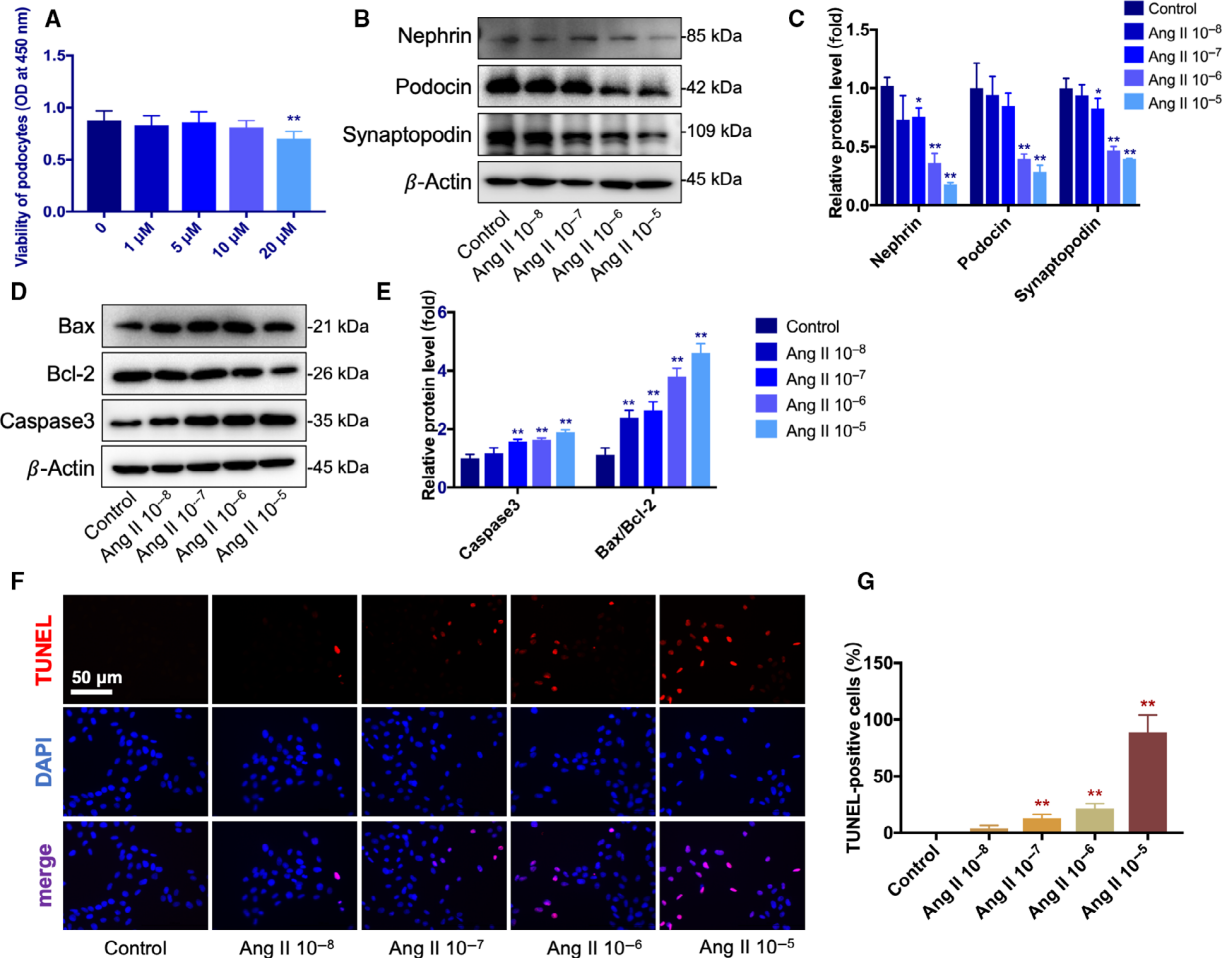
Effect of curcumin on podocyte viability and effect of Ang II on podocyte‐specific proteins and apoptosis. (A) Cell viability was determined using a CCK‐8 assay. Twenty micromolar curcumin slightly reduced the viability of podocytes. (B, C) The levels of nephrin, podocin, and synaptopodin were detected using western blotting. Ang II dose‐dependently reduced the levels of nephrin and podocin. (D, E) The levels of Bax, Bcl‐2, and caspase‐3 were detected using western blotting. Ang II dose‐dependently increased the expression of the Bax, Bcl‐2, and caspase‐3. (F, G) The effect of Ang II on cell apoptosis was detected using TUNEL staining. Scale bar = 50 μm. From 10^−8^ to 10^−5^ mol·L^−1^, TUNEL‐positive cells were dose‐dependently increased. Data are expressed as the mean ± standard deviation (*n* = 3). Data were analyzed by one‐way ANOVA with Bonferroni's test for multiple comparisons (A, C, E, G). *P < 0.05 and **P < 0.01 *vs*. the control.

To determine the noncytotoxic concentration of curcumin in podocytes, we evaluated their viability using a CCK‐8 assay. Cells were exposed to different concentrations (0, 1, 5, 10, and 20 μm) of curcumin for 48 h. As shown in Fig. 1, 20 μm curcumin slightly reduced the viability of podocytes after 48 h of treatment. Therefore, we chose 1, 5, and 10 μm as a concentration gradient for subsequent experiments (Fig. 1A).

### Effect of Ang II on podocyte‐specific proteins and apoptosis

We examined the effects of Ang II on the reduction of podocyte‐specific proteins in cultured podocytes. Podocytes were treated with various concentrations of Ang II (10^−8^ to 10^−5^ mol·L^−1^) for 48 h. As shown in Fig. 1, Ang II reduced the levels of nephrin, podocin, and synaptopodin in a dose‐dependent manner (Fig. 1B,C). To determine whether Ang II could exert a pro‐apoptotic effect on cultured podocytes, we treated podocytes with various concentrations of Ang II (10^−8^ to 10^−5^ mol·L^−1^) for 48 h. We found angiotensin II dose‐dependently increased the expression of the Bax, Bcl‐2, and caspase‐3 proteins (Fig. 1D,E). The results of TUNEL staining showed that TUNEL‐positive cells were dose‐dependently increased in the Ang II‐treated groups (Fig. 1F,G). Based on these data and previous studies, the cells were treated with 10^−6^ mol·L^−1^ Ang II for 48 h in subsequent experiments.

### Curcumin attenuated Ang II‐induced podocyte injury

**Fig. 2:**
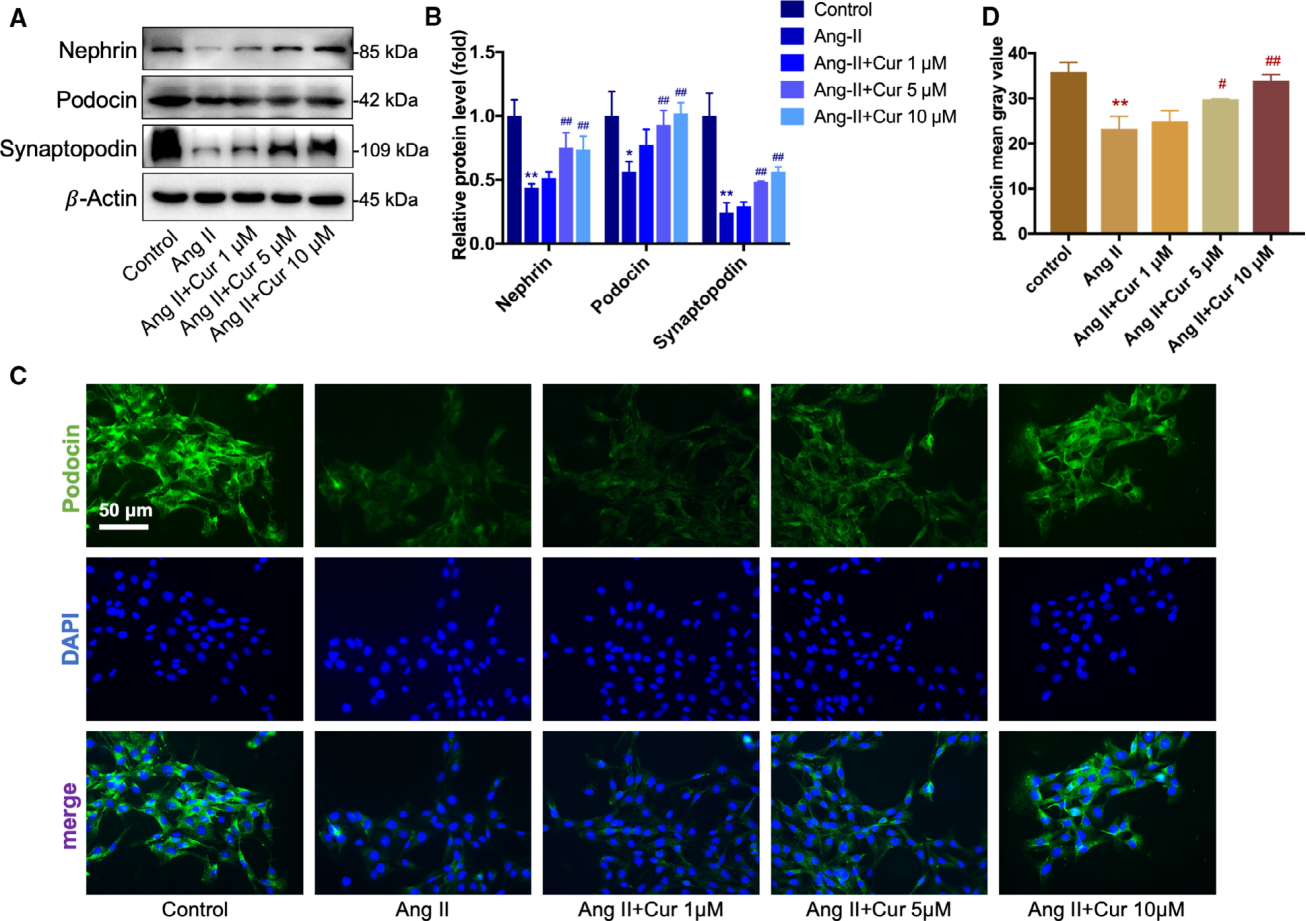
Curcumin attenuated Ang II‐induced podocyte injury *in vitro*. (A, B) Western blotting analysis of nephrin, podocin, and synaptopodin. Curcumin dose‐dependently reversed the decrease in the expression of nephrin, podocin, and synaptopodin induced by Ang II. (C, B) Podocin was stained using immunofluorescence, and the nuclei were counterstained using DAPI (×400). Scale bar = 50 μm. Ang II‐induced decrease in podocin was dose‐dependently attenuated by curcumin. Data are expressed as the mean ± standard deviation (*n* = 3). Data were analyzed by one‐way ANOVA with Bonferroni's test for multiple comparisons (B, D). *P < 0.05 and **P < 0.01 *vs*. the control. ^#^ *P* < 0.05 and ^##^ *P* < 0.01 *vs*. the Ang II.

**Fig. 3:**
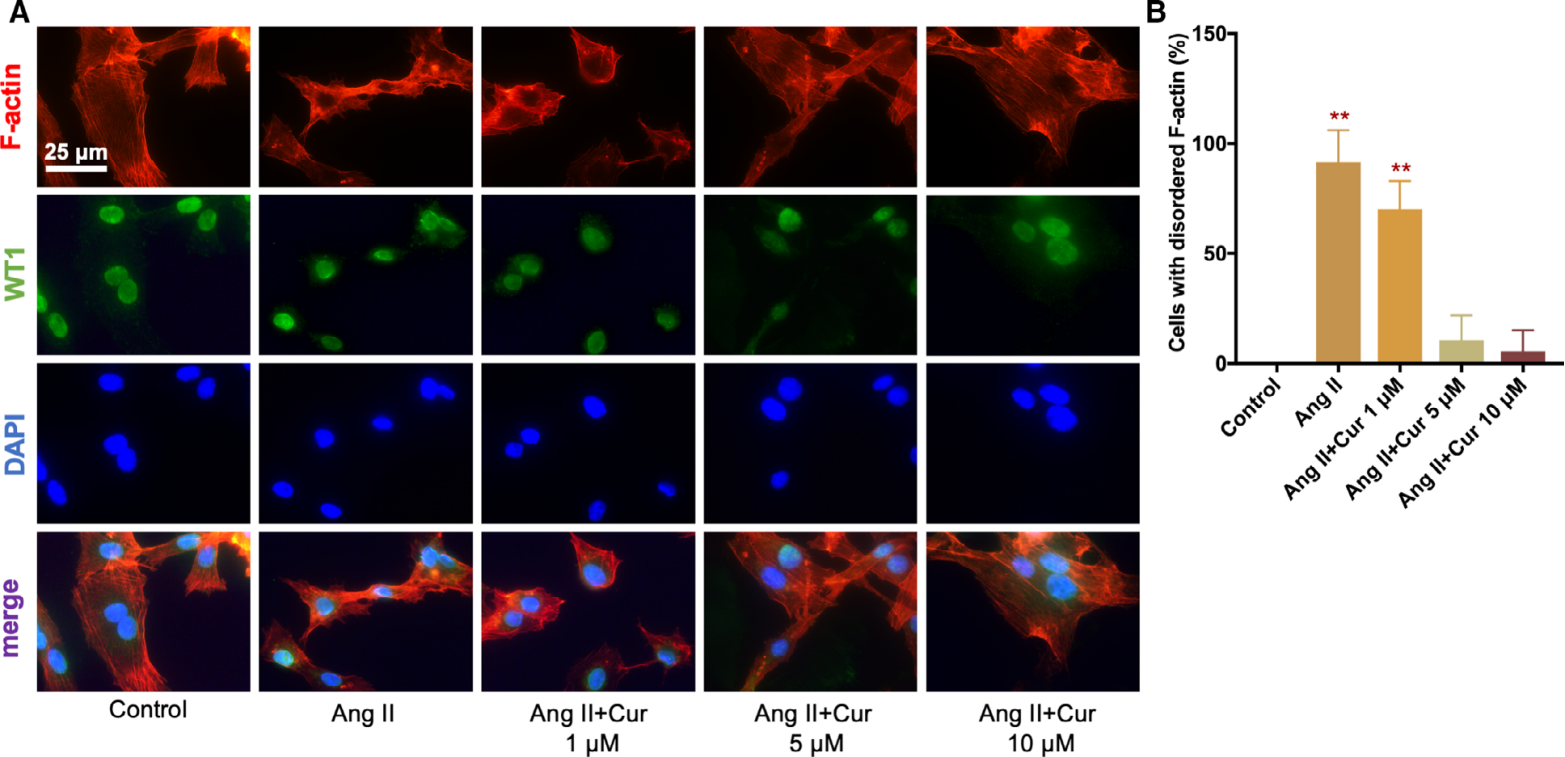
Curcumin reversed Ang II‐induced rearrangement of F‐actin in podocytes. (A, B) F‐actin and WT1 were stained using immunofluorescence while the nuclei were counterstained using DAPI (×1000). Scale bar = 25 μm. Curcumin pretreatment prevented Ang II‐induced cortical F‐actin ring formation and stress fiber attenuation in a dose‐dependent manner. Data are expressed as the mean ± standard deviation (*n* = 3). Data were analyzed by one‐way ANOVA with Bonferroni's test for multiple comparisons (B). *P < 0.05 and **P < 0.01 *vs*. the control. ^#^ *P* < 0.05 and ^##^ *P* < 0.01 *vs*. the Ang II.

Podocyte‐specific proteins are crucial structures in foot processes of podocytes, and their injuries play key roles in the pathogenesis of CKD. We examined nephrin, podocin, and synaptopodin using western blotting in podocytes that were pretreated with various concentrations (1, 5, and 10 μm) of curcumin (Cur) for 1 h and then incubated with Ang II for 48 h. The results showed that pretreating podocytes with curcumin reduced Ang II‐induced decreases in nephrin, podocin, and synaptopodin levels in a dose‐dependent manner (Fig. 2A,B). The result of immunofluorescence staining with podocin was consistent with those of western blotting (Fig. 2C,D). Meanwhile, exposure of podocytes to Ang II resulted in a marked change of the expression and arrangement of F‐actin, including cortical F‐actin ring formation and stress fiber attenuation. Curcumin treatment prevented these changes in a dose‐dependent manner (Fig. 3A,B).

### Curcumin attenuated Ang II‐enhanced apoptosis in podocyte

**Fig. 4:**
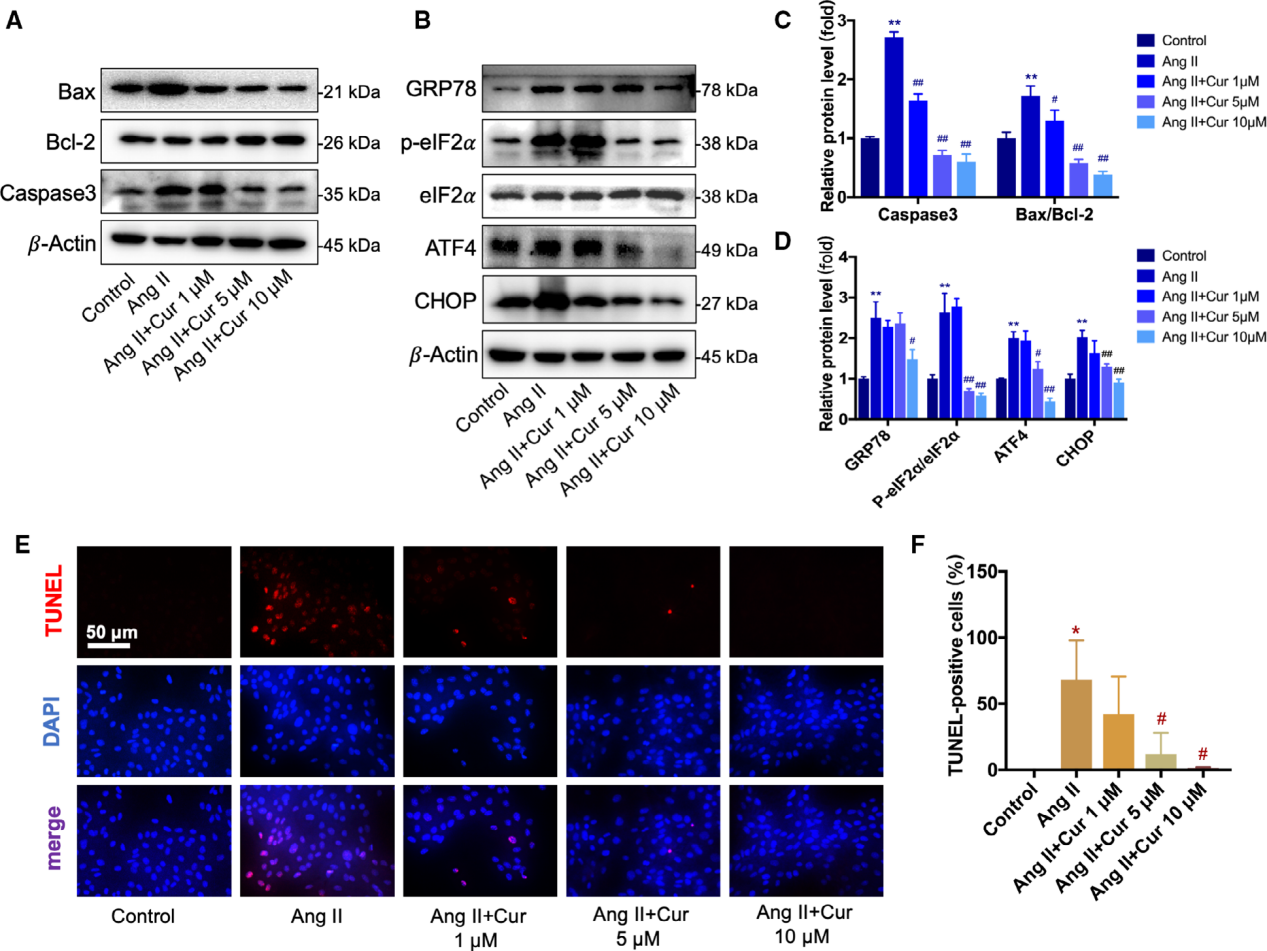
Curcumin attenuated Ang II‐enhanced apoptosis and ER stress in podocytes. (A, C) Western blotting analysis of Bax, Bcl‐2, and caspase‐3. Curcumin markedly attenuated Ang II‐upregulated expression of Bax, Bcl‐2, and caspase‐3. (B, D) The expression level of ER stress‐related proteins including GRP78, ATF4, p‐eIF2α, eIF2α, and CHOP was detected using western blotting. Ang II‐upregulated ER stress‐related proteins were downregulated by curcumin treatment. (E, F) Cell apoptosis was observed using a TUNEL assay, and the cells were examined under a fluorescence microscope (×400). Scale bar = 50 μm. Ang II increased apoptosis in podocytes compared with that in the control group, and 5 and 10 μm curcumin remarkably reversed the change. Data are expressed as the mean ± standard deviation (*n* = 3). Data were analyzed by one‐way ANOVA with Bonferroni's test for multiple comparisons (C, D, F). *P < 0.05 and **P < 0.01 *vs*. the control. ^#^ *P* < 0.05 and ^##^ *P* < 0.01 *vs*. the Ang II.

Apoptotic cell death is believed as one of the manifestations of podocyte injury in CKD. Thus, we next examined Bax, Bcl‐2, and caspase‐3 in podocytes. Podocytes were incubated with various concentrations (1, 5, and 10 μm) of curcumin for 1 h before treatment with Ang II (10^−6^ mol·L^−1^) for 48 h. We found that exposing podocytes to Ang II‐induced significant podocyte apoptosis, whereas curcumin markedly attenuated Ang II‐induced podocyte apoptosis in a concentration‐dependent manner (Fig. 4A,C). Moreover, the results of TUNEL staining were consistent with those of western blotting (Fig. 4E,F).

### Curcumin decreased the Ang II‐induced podocyte injury and apoptosis by attenuating ER stress

**Fig. 5:**
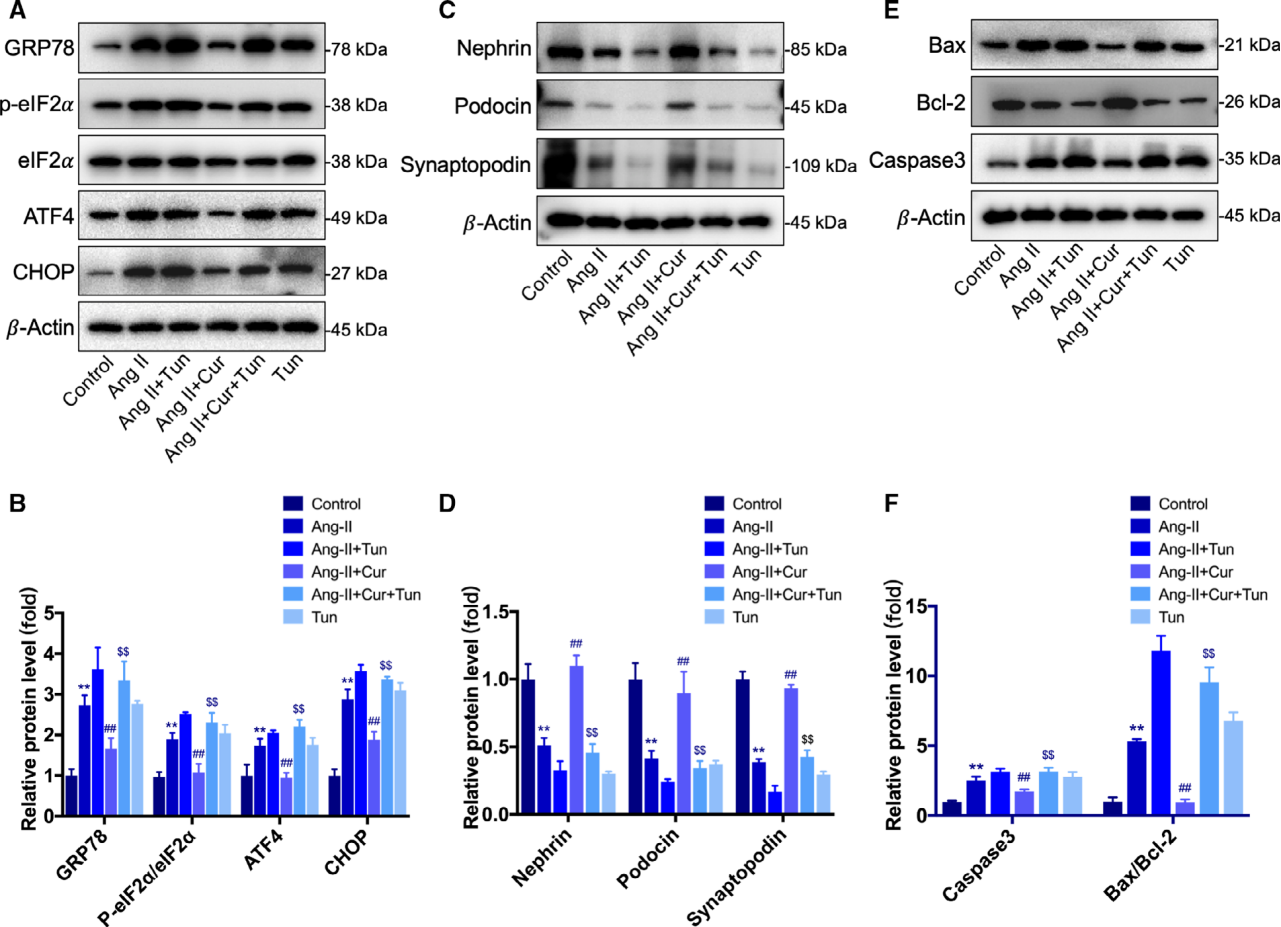
Curcumin decreased the Ang II‐induced podocyte injury and apoptosis by attenuating ER stress. (A, B) Western blotting analysis of ER stress‐related proteins. Tunicamycin reactivated ER stress, which was suppressed by curcumin. (C, D) Western blotting analysis of podocyte‐specific proteins. Compared with the Ang II+Cur group, the protein levels of nephrin, podocin, and synaptopodin were reduced in the Ang II+Cur+Tun group. (E, F) Western blotting analysis of apoptosis‐related proteins. Compared with the Ang II+Cur group, the protein levels of Bax, Bcl‐2, and caspase‐3 were increased in the Ang II+Cur+Tun group. Data are expressed as the mean ± standard deviation (*n* = 3). Data were analyzed by one‐way ANOVA with Bonferroni's test for multiple comparisons (B, D, F). *P < 0.05 and **P < 0.01 *vs*. the control. ^#^ *P* < 0.05 and ^##^ *P* < 0.01 *vs*. the Ang II. ^$^ *P* < 0.05 and ^$$^ *P* < 0.01 *vs*. the Ang II+Cur.

We further investigated whether prolonged ER stress is involved in the protective role of curcumin on the Ang II‐treated podocytes. Western blotting was used to measure the levels of ER stress‐related proteins GRP78, ATF4, eIF2α, p‐eIF2α, and CHOP. As shown in Fig. 3, the expression levels of GRP78, ATF4, p‐eIF2α, and CHOP were obviously increased in the Ang II‐treated group compared with those in the control group and were significantly inhibited in the curcumin 5 and 10 μm groups compared with those in the Ang II‐treated group (Fig. 4B,D). Then, we added the ER stress pathway activator tunicamycin (Tun, 5 μg·mL^−1^) to Ang II (10^−6^ mol·L^−1^) + Cur (10 μm) group. We observed the suppressed ER stress was reactivated (Fig. 5A,B), while curcumin's protective effects on podocyte injury and apoptosis were reversed (Fig. 5C–F). These results suggested that the protective effects of curcumin in podocytes function, at least partially, by inhibiting the ER stress pathway.

## Discussion

In the present study, we confirmed that curcumin could attenuate Ang II‐downregulated podocyte‐specific proteins and that Ang II enhanced podocyte apoptosis using an Ang II‐induced podocyte injury model. Moreover, we found the podocyte protective effect of curcumin could act via inhibiting ER stress.

Previous studies have confirmed that elevated Ang II levels can induce podocyte injury and apoptosis [2, 4, 19]; therefore, we used Ang II to establish a podocyte injury model. At the same time, we found that curcumin pre‐intervention can alleviate Ang II‐induced podocyte injury and apoptosis, and has a certain podocyte protective effect. Recent studies also supported the podocyte protective effect of curcumin: Curcumin could protect against fructose‐induced glomerular podocyte injury and proteinuria [20]. Sun *et al*. [21] found that curcumin attenuated high glucose‐induced podocyte apoptosis *in vitro* and diabetic nephropathy *in vivo*, partly through regulating the functional connections between caveolin‐1 phosphorylation and reactive oxygen species. However, there has been no relevant study about the protective capacity of curcumin on Ang II‐induced podocyte dysfunction. Therefore, the present study is the first to explore the protective effect of curcumin on podocyte injury induced by Ang II. Several studies observed that in rat kidney tissue, mouse atherosclerotic lesion tissue, adult mouse cardiomyocytes (HL‐1), and vascular smooth muscle cells, curcumin could reduce several injury factor‐mediated upregulation of AT1R [22, 23, 24, 25]. We speculated that the podocyte protective effect of curcumin may also be related to this mechanism. However, there are few studies related to how does curcumin regulate AT1R, and the specific molecular mechanism is worthy of further exploration. Abnormally sustained elevated Ang II is a key factor in podocyte injury, proteinuria, and the occurrence and progression of CKD; therefore, the present study might provide a new direction for the treatment of CKD via the RAS system.

Our data showed that the levels of ER stress‐related proteins were significantly increased in the Ang II‐treated group compared with the control group, which suggested that Ang II could induce ER stress. These results were consistent with those of previous studies [12, 26, 27, 28]. Moreover, the therapeutic effect of curcumin is associated with ER stress [29]. Afrin *et al*. [30] found that curcumin preserves renal function, probably by attenuating ER stress‐mediated mitogen‐activated protein kinase signaling. Our results further confirmed the anti‐ER stress effect of curcumin. But how the curcumin interacts with endoplasmic reticulum? Some studies suggested that this is related to the high antioxidant potential of curcumin. Curcumin inhibited furazolidone‐induced ER stress in human hepatocyte L02 cells through activation of the Nrf2/HO‐1 pathway [31]. Similarly, curcumin also attenuated oxidative stress‐induced endoplasmic reticulum‐dependent apoptosis of splenocytes in STZ‐induced diabetic rats [32]. Studies also showed that in human umbilical vein endothelial cells, curcumin‐activated compensative autophagy could maintain ER protein homeostasis by degradation of damaged and aggregated proteins [33]. Furthermore, curcumin could inhibit the upregulation of intracellular Ca^2+^; this may also be one of its potential mechanisms to inhibit ER stress [32].

In conclusion, the present study demonstrated that curcumin could attenuate Ang II‐induced podocyte injury and apoptosis, at least partly by inhibiting ER stress. Therefore, curcumin might be a novel therapeutic agent for CKD.

## Conflict of interest

The authors declare no conflict of interest.

## Author contributions

NY, JN, and YG conceived and designed the study. YY, YL, and LL acquired the data. NY, QW, and LY analyzed and interpreted the data. NY prepared the manuscript.

## Data Availability

Raw data are available from the corresponding author upon reasonable request.
